# Transfer of microRNA-25 by colorectal cancer cell-derived extracellular vesicles facilitates colorectal cancer development and metastasis

**DOI:** 10.1016/j.omtn.2020.11.018

**Published:** 2020-11-26

**Authors:** Shanchao Wang, Zeyan Zhang, Qianfu Gao

**Affiliations:** 1 Department of Anorectal, Linyi People’s Hospital, Linyi 276003, Shandong Province, P.R. China

**Keywords:** colorectal cancer, extracellular vesicles, microRNA-25, sirtuin 6, lin-28 homolog B, neuropilin-1, metastasis

## Abstract

Cancer cell-derived extracellular vesicles (EVs) have been reported to promote the progression of colorectal cancer (CRC), although the regulatory mechanism remains uncharacterized. In this study, we investigated the role of microRNA-25 (miR-25)/sirtuin 6 (SIRT6) in the contribution of EVs derived from CRC cells to progression of CRC. In a co-culture system with EVs from HCT116 and NCM460 cells, the viability, migratory, and invasive properties of SW480 and SW620 cells were evaluated by cell counting kit-8 (CCK-8) and Transwell assays. Luciferase, chromatin immunoprecipitation (ChIP), and RNA immunoprecipitation (RIP) assays were conducted to verify the interaction among miR-25, SIRT6, lin-28 homologB (Lin28b), and neuropilin-1 (NRP-1). It was established that HCT116 cell-derived EVs promoted the malignant properties of SW480 cells and SW620 cells by delivering miR-25. SIRT6 was targeted by miR-25, whereas SIRT6 inhibited NRP-1 through downregulation of Lin28b. The tumor-bearing nude mouse experiments substantiated that HCT116 cell-derived EVs transferred miR-25 to facilitate tumor formation and metastasis by inhibiting SIRT6. In summary, our study clarifies the involvement of miR-25-targeted SIRT6 inhibition and SIRT6-mediated inhibition of the Lin28b/NRP-1 axis in CRC cell-derived EVs to CRC progression and metastasis.

## Graphical Abstract

**Figure:**
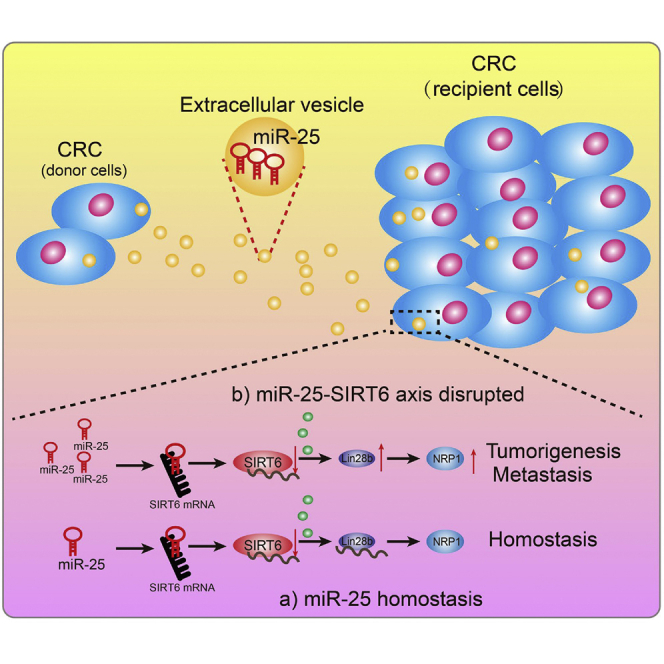

## Introduction

Colorectal cancer (CRC) is one of the leading causes of cancer-related death globally, and the mortality of patients with CRC ranks fifth in China according to cancer statistics in 2015.^1^^,^^2^ Although improved screening and surveillance make great contributions to the early detection of CRC, minimal progress has been achieved in mitigating the devastating impact of CRC metastasis to the liver.^3^ Also, lung or liver metastases of CRC remain a major obstacle in clinical therapeutics.^4^ Thus, more effective therapies for CRC are urgently needed. Extracellular vesicles (EVs) are crucial intercellular communication modulators that play important roles in the processes of infiltration, metastasis, and immune tolerance during tumorigenesis.^5^^,^^6^ These effects of EVs are part of the general capacity to transmit a diverse array of bioactive signaling, surface receptors, protein-coding mRNAs, and microRNAs (miRNAs, or miRs).^7^

miRNAs are small and non-coding regulatory RNAs, which have emerged as valuable biomarkers and therapeutic targets for CRC.^8^ For example, miR-25 is highly expressed in CRC and is a marker of poor prognosis of patients with CRC.^9^ Intriguingly, a major type of EVs, namely the exosomes, can transfer miR-25 from CRC cells to endothelial cells to promote the occurrence and metastasis of CRC.^10^ However, the mechanism underlying this process remains largely unexplored.

Sirtuin 6 (SIRT6) is a member of the sirtuin family of NAD(+)-dependent deacetylases, which plays significant role in the regulation of metabolism, inflammation, and aging.^11^ In the present context, SIRT6 protein acts as a suppressor of colon tumorigenesis, which suppresses pancreatic cancer through control of the zinc finger protein Lin28b, which drives the growth and survival of SIRT6-deficient pancreatic cancer.^12^ The decreased expression of SIRT6 is reportedly related to the progression of CRC, such that SIRT6 has potential as a diagnostic and prognostic biomarker for CRC.^13^ More recent research shows that SIRT6 can mediate the progression of colon cancer by functioning as a novel direct transcriptional target of FoxO3a.^14^ Furthermore, the function of SIRT6 is related to miRNAs, since it can function as a target of miRNAs. For example, miR-351-5p can aggravate intestinal ischemia-reperfusion injury through inhibiting SIRT6.^15^ In our study, our interrogation of the starBase 3.0 software predicted SIRT6 to be a target of miR-25, leading us to speculate that miR-25 in EVs derived from CRC cells may target SIRT6 to affect colon tumorigenesis.

SIRT6 can bind to the promoter region of the lin-28 homolog B (Lin28b) gene and inhibit the expression of Lin28b through deacetylation, in which SIRT6 actively co-represses Myc-dependent transcription in human and murine pancreatic ductal adenocarcinomas specifically at the Lin28b locus, through deacetylation of the H3K56 and H3K9 chromatin marks.^16^ Lin28b gain-of-function promotes tumor cell migration and tumor relapse in CRC.^17^ Lin28b, as an RNA binding protein, can bind to the 3′ untranslated region (UTR) of neuropilin 1 (NRP-1) to stabilize its mRNA and promote the expression of NRP-1.^18^ Also, NRP-1 functions as a facilitator in the angiogenic, migratory, and invasive properties of tumor cells^19^ and can promote liver and lung metastasis in CRC.^20^ In view of the above findings, we aimed in this study to explore whether miR-25, SIRT6, Lin28b, and NRP-1 are potential molecular participants responsible for the promotion of EVs derived from CRC cells to CRC metastasis.

## Results

### Enrichment of miR-25 in CRC cell-derived EVs and its transfer by EVs

**Figure 1:**
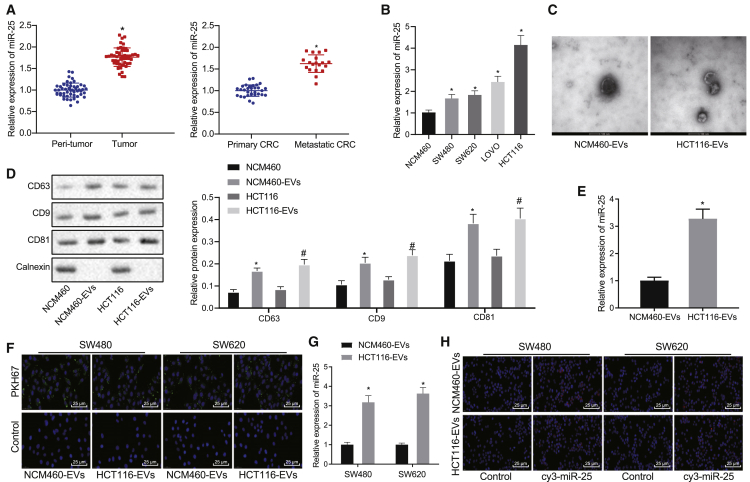
miR-25 was highly expressed in EVs derived from CRC cells, and these EVs could mediate the transfer of miR-25 (A) qRT-PCR was used to detect the expression of miR-25 in CRC tissues (n = 50), adjacent non-tumor tissues, and cancer tissues from patients with metastatic CRC (n = 19) and without primary CRC (n = 31); ∗p < 0.05 versus peri-tumor group or primary CRC group. (B) The expression of miR-25 in CRC cell lines and normal colon epithelial cells detected by qRT-PCR. ∗p < 0.05 versus NCM460 group. (C) The morphology of EVs observed by transmission electron microscopy (scale bar, 100 nm). (D) EV-specific surface marker proteins examined by western blot assay. ∗p < 0.05 versus NCM460 group, #p < 0.05 versus HCT116 group. (E) Expression of miR-25 in EVs extracted from NCM460 and HCT116 detected by qRT-PCR, respectively. ∗p < 0.05 versus NCM460-EVs group. (F) PKH67-labeled EVs were incubated with SW480 and SW620 cells for 24 h to observe the internalization of EVs (400×) by fluorescence microscope. (G) Expression of miR-25 in SW480 and SW620 cells after co-culture with EVs from NCM460 and HCT116 cells detected by qRT-PCR. ∗p < 0.05 versus NCM460-EVs group. (H) Cy3-labeled miR-25-mimic was transfected into NCM460 and HCT116 cells, EVs extracted from which were then co-cultured with SW480 and SW620 cells to observe the transfer of Cy3-labeled miR-25-mimic (400×) by fluorescence microscope. The results were measurement data and expressed as mean ± SD. Paired t test was used to compare cancer tissues and adjacent non-tumor tissues, unpaired t test was used for comparison between other two groups, and one-way analysis of variance was used for comparison among multiple groups.

In the first phase of the study, clinical CRC tissues were collected and analyzed by quantitative reverse-transcriptase polymerase chain reaction (qRT-PCR), which showed higher miR-25 expression in cancer tissues than in the matched non-cancerous tissues, and that miR-25 expression was higher in cancer tissues of patients with metastasis than in those from patients without metastasis (Figure 1A). Next, we further examined miR-25 expression in CRC cell lines (HCT116, SW480, SW620, and LOVO) and normal colon cells (NCM460 cells). The results (Figure 1B) showed higher miR-25 expression in CRC cells than in normal cells, with highest miR-25 expression in HCT116 cells. HCT116, SW480, and SW620 cells were selected for the subsequent experiments.

To verify whether the miR-25 in EVs derived from CRC cells could affect the metastatic characteristics of cancer cells, we extracted EVs from NCM460 and HCT116 cells. Transmission electron microscopy (TEM) observation showed that the EVs were cup- or spherical-shaped^10^ (Figure 1C). Western blot assay detection results (Figure 1D) showed enrichment of CD63, CD81, and CD9 in EVs, indicating the successful separation of EVs. Further qRT-PCR detection revealed an upregulation of miR-25 expression in HCT116-EVs relative to NCM460-EVs (Figure 1E). To observe the internalization of EVs by SW480 and SW620 cells, we labeled EVs with PKH67 and incubated them with SW480 and SW620 cells for 24 h. Fluorescence microscopic observation presented green fluorescence in PKH67-labeled cells, indicating that SW480 and SW620 cells could indeed internalize HCT116-EVs and NCM460-EVs (Figure 1F). The NCM460-EVs or HCT116-EVs were incubated with SW480 and SW620 cells. qRT-PCR results exhibited that HCT116-EV treatment increased the miR-25 expression in SW480 and SW620 cells compared to NCM460-EVs (Figure 1G). To further confirm that miR-25 was transferred by EVs, Cy3 was used to label miR-25 mimic, which was transfected into HCT116 and NCM460 cells. Subsequently, EVs were extracted from the transfected SW480 and SW620 cells. Fluorescence microscopy observation revealed significant red fluorescence in the Cy3-labeled cells (Figure 1H), suggesting the successful transfer of miR-25 via EVs. Therefore, we concluded that miR-25 was highly expressed in EVs secreted from CRC cells and could be transferred to CRC cells through EVs.

### miR-25 transferred by CRC cell-derived EVs promotes the viability and migration of CRC cells

**Figure 2:**
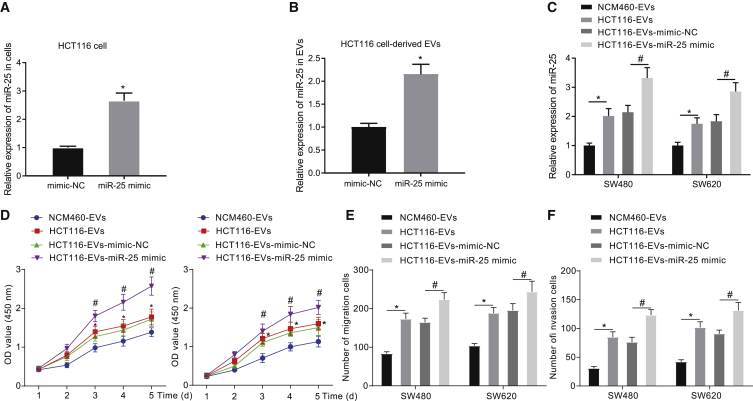
The malignant phenotypes of CRC cells could be strengthened by miR-25 from CRC cell-derived EVs (A) Expression of miR-25 in HCT116 cells transfected with miR-25 mimic detected by qRT-PCR. ∗p < 0.05 versus mimic-NC transfected cells. (B) Expression of miR-25 in EVs derived from HCT116 cells transfected with miR-25 mimic detected by qRT-PCR. ∗p < 0.05 versus EVs derived from mimic-NC transfected cells. (C) Expression of miR-25 in the SW480 and SW620 cells in the co-culture system detected by qRT-PCR. ∗p < 0.05 versus cells co-cultured with NCM460-EVs, #p < 0.05 versus cells co-cultured with HCT116-EVs-mimic-NC. (D) Cell viability in the co-culture system detected by CCK-8 assay. (E) Cell migration in the co-culture system detected by Transwell assay. (F) Cell invasion in the co-culture system detected by Transwell assay. In (C)–(F), SW480 and SW620 cells were co-cultured with NCM460-EVs, HCT116-EVs, HCT116-EVs-mimic-NC, and HCT116-EVs-miR-25 mimic. ∗p < 0.05 versus cells co-cultured with NCM460-EVs, #p < 0.05 versus cells co-cultured with HCT116-EVs-mimic-NC. The results were measurement data expressed as mean ± SD. The data between the two groups of data were analyzed by unpaired t test, data between multiple groups were analyzed by one-way analysis of variance, and data between two groups at different time points were analyzed by two-way analysis of variance.

To further examine the effect of EV-shuttled miR-25 on the functions of CRC cells, miR-25 was overexpressed in HCT116 cells by transfection with miR-25 mimic, and EVs were later extracted from the transfected HCT116 cells. qRT-PCR suggested an upregulation of miR-25 in HCT116 cells by miR-25 mimic and in their derived EVs (Figures 2A and 2B). SW480 and SW620 cells were co-cultured with NCM460-EVs, HCT116-EVs, HCT116-EVs-mimic-negative control (NC), and HCT116-EVs-miR-25 mimic, respectively. As shown by qRT-PCR (Figure 2C), an increased miR-25 expression was observed in the cells after co-culture with HCT116-EVs relative to those co-cultured with NCM460-EVs. Furthermore, co-culture with HCT116-EVs-miR-25 mimic led to higher expression of miR-25 than after HCT116-EVs-mimic-NC treatment. The results of functional experiments showed that co-culture with HCT116-EVs promoted the proliferative, migratory, and invasive properties of SW480 and SW620 cells relative to NCM460-EVs, while co-culture with HCT116-EVs-miR-25 mimic contributed to an enhancement of viability, migratory, and invasive abilities (Figures 2D–2F). The above results indicated that CRC cell-derived EVs could promote malignant phenotypes of tumor cells by delivering miR-25.

### SIRT6 is the target gene of miR-25

**Figure 3:**
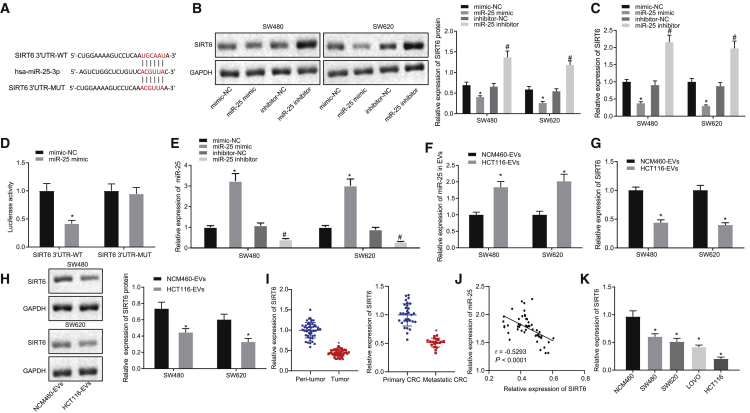
SIRT6 is negatively targeted by miR-25 (A) The starBase 3.0 online website prediction of the specific binding sites between SIRT6 and miR-25 and the targeted mutation sites. (B) SIRT6 protein expression detected by western blot analysis after SW480 and SW620 cells were transfected with miR-25 mimic or inhibitor. ∗p < 0.05 versus mimic-NC transfected cells. #p < 0.05 versus inhibitor-NC transfected cells. (C) SIRT6 expression detected by qRT-PCR after SW480 and SW620 cells were transfected with miR-25 mimic or inhibitor. ∗p < 0.05 versus mimic-NC transfected cells. #p < 0.05 versus inhibitor-NC transfected cells. (D) Dual-luciferase reporter gene assay for validation of the binding of miR-25 to SIRT6. ∗p < 0.05 versus mimic-NC transfected cells. (E) The miR-25 expression in SW480 and SW620 cells transfected with miR-25 mimic and miR-25 inhibitor detected by qRT-PCR, ∗p < 0.05 versus mimic-NC transfected cells, #p < 0.05 versus inhibitor-NC transfected cells. (F) SIRT6 mRNA expression in SW480 and SW620 cells transfected with miR-25 mimic and miR-25 inhibitor detected by qRT-PCR. ∗p < 0.05 versus mimic-NC transfected cells, #p < 0.05 versus inhibitor-NC transfected cells. (G) SIRT6 expression in SW480 and SW620 cells after co-culture with EVs detected by qRT-PCR. ∗p < 0.05 versus NCM460-EVs co-cultured cells. (H) Western blot assay detection of the expression of SIRT6 protein in SW480 and SW620 cells co-cultured with EVs. ∗p < 0.05 versus NCM460-EVs transfected cells. (I) the expression of SIRT6 in CRC tissues (n = 50), adjacent non-tumor tissues (n = 50), and cancer tissues of patients with primary CRC (n = 31) and metastatic CRC (n = 19) detected by qRT-PCR. ∗p < 0.05 versus peri-tumor group or primary CRC group. (J) Pearson’s correlation analysis of correlation between SIRT6 and miR-25 expression in cancer tissues. (K) The expression of SIRT6 in CRC cell line and normal colon cell NCM460 detected by qRT-PCR. ∗p < 0.05 versus NCM460 group. The results were measurement data expressed as mean ± SD. Paired t test was used to compare cancer tissues and adjacent tissues, unpaired t test was used for comparison between the remaining two groups, and one-way analysis of variance was used for comparison among multiple groups.

To further study the molecular mechanism of miR-25 influencing CRC, the target genes of miR-25 were predicted through the starBase 3.0 website, which reported the potential binding sites between miR-25 and SIRT6 3′UTR (Figure 3A). The SIRT6 expression was determined at protein and mRNA levels by western blot assay (Figure 3B) and qRT-PCR (Figure 3C). miR-25 mimic transfection led to increased miR-25 expression and decreased SIRT6 mRNA and protein levels. Conversely, miR-25 inhibitor transfection resulted in downregulated miR-25 expression and upregulated SIRT6 mRNA and protein levels. The dual-luciferase reporter gene assay verified the binding relationship that the luciferase activity of SIRT6 3′UTR-wild type (WT) was reduced by miR-25 mimic, but the luciferase activity of SIRT6 3′UTR-mutant type (MUT) was unaffected (Figure 3D). After transfection with miR-25 mimic and inhibitor in SW480 and SW620 cells, the miR-25 expression was detected by qRT-PCR (Figure 3E). After treatment of SW480 and SW620 cells with EVs, miR-25 expression in the cells was detected by qRT-PCR (Figure 3F), and the intracellular SIRT6 level was as detected by qRT-PCR (Figure 3G) and western blot assay (Figure 3H). The results showed that co-culture with HCT116-EVs resulted in upregulated miR-25 expression and downregulated SIRT6 levels as compared to co-culture with NCM460-EVs. qRT-PCR was utilized to quantify SIRT6 expression in clinical samples, which showed lower SIRT6 expression in cancer tissues than in adjacent non-tumor tissues. Furthermore, SIRT6 expression in cancer tissues of patients with metastatic CRC was also lower than that in cancer tissues of patients with primary CRC (Figure 3I). Pearson’s correlation analysis exhibited that SIRT6 expression was negatively correlated with miR-25 (Figure 3J). Consistent with the expression pattern in tissues, expression of SIRT6 in CRC cells was lower than that in normal cells NCM460 (Figure 3K).

### EV-shuttled miR-25 inhibits SIRT6 expression to promote the malignant properties of CRC cells

**Figure 4:**
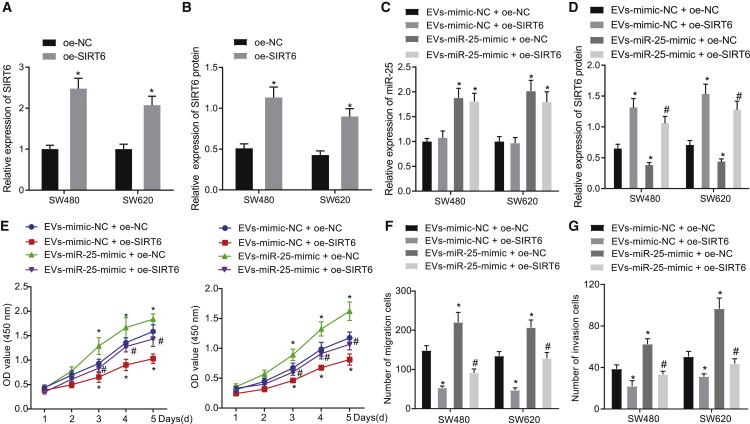
miR-25 carried by CRC cell-derived EVs could promote the malignant properties of CRC cells through inhibiting SIRT6 expression (A) SIRT6 at mRNA level in SW480 and SW620 cells after transfection with OE-SIRT6 detected by qRT-PCR. ∗p < 0.05 versus OE-NC-transfected cells. (B) SIRT6 at protein level in SW480 and SW620 cells after transfection with OE-SIRT6 detected by western blot assay. ∗p < 0.05 versus OE-NC-transfected cells. (C) miR-25 expression in SW480 and SW620 cells detected by qRT-PCR. (D) SIRT6 protein expression in SW480 and SW620 cells detected by western blot assay. (E) SW480 and SW620 cell viability detected by CCK-8 assay. (F) SW480 and SW620 cell migration detected by Transwell assay. (G) SW480 and SW620 cell invasion detected by Transwell assay. In (C)–(G), SW480 and SW620 cells were transfected with OE-NC/OE-SIRT6 and then co-cultured with the EVs-mimic-NC/EVs-miR-25-mimic. ∗p < 0.05 versus EVs-mimic-NC + OE-NC group, #p < 0.05 versus EVs-miR-25-mimic + OE-NC group. The results were measurement data expressed as mean ± SD, with comparison of data between two groups conducted using unpaired t test, comparison of data among multiple groups conducted using one-way analysis of variance, and, in (E), comparison between two groups at different time points conducted using two-way analysis of variance.

To study the interaction of miR-25 and SIRT6 in the implications of EVs on CRC cells, SIRT6 was overexpressed in CRC cells SW480 and SW620, which were co-cultured with the EVs-miR-25 mimic. The overexpression efficiency was tested by qRT-PCR and western blot assay, which displayed a successful upregulation of SIRT6 by overexpression (OE)-SIRT6 (Figures 4A and 4B). qRT-PCR (Figure 4C) and western blot assay (Figure 4D) also exhibited that the co-culture of EVs-miR-25-mimic increased the miR-25 expression and suppressed the SIRT6 expression in the SW480 and SW620 cells transfected with OE-NC. Treatment with OE-SIRT6 elevated the SIRT6 expression in the SW480 and SW620 cells co-cultured with EVs-mimic-NC and rescued the SIRT6 expression inhibited by the co-culture of EVs-miR-25-mimic.

By detecting changes in cellular functions, we found that co-culture of EVs-miR-25-mimic accelerated the proliferating, migratory, and invasive potentials of SW480 and SW620 cells transfected with OE-NC, while OE-SIRT6 inhibited those malignant functions in the co-culture system of EVs-mimic-NC. The aforementioned enhancement of malignant abilities of SW480 and SW620 cells by EVs-miR-25-mimic were reduced by restoration of SIRT6 expression (Figures 4E–4G). Hence, CRC cell-derived EVs transferred miR-25 into CRC cells, which downregulated SIRT6 expression, thereby accelerating the progression of CRC *in vitro*.

### SIRT6 regulates the Lin28b/NRP-1 axis to inhibit the viability and migration of CRC cells

**Figure 5:**
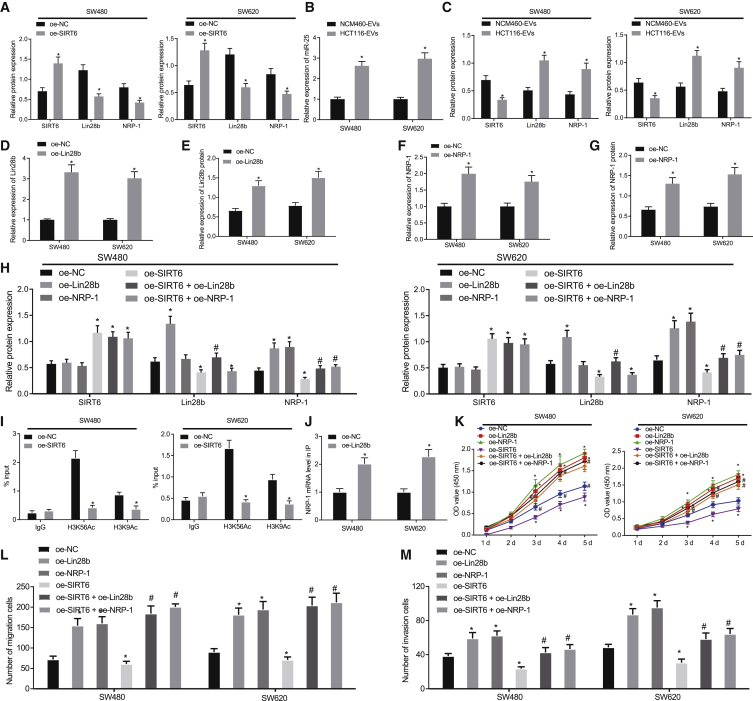
SIRT6 inhibits the viability and migration of CRC cells through regulating Lin28b/NRP-1 axis (A) Western blot assay of the protein expression of SIRT6, NRP-1, and Lin28b in the SW480 and SW620 cells transfected with OE-SIRT6. ∗p < 0.05 versus OE-NC-transfected cells. (B) qRT-PCR detection of the expression of miR-25 in the SW480 and SW620 cells co-cultured with NCM460-EVs/HCT116-EVs. ∗p < 0.05 versus NCM460-EVs-treated cells. (C) Western blot assay of the protein expression of SIRT6, NRP-1, Lin28b, in the SW480 and SW620 cells co-cultured with NCM460-EVs/HCT116-EVs. ∗p < 0.05 versus NCM460-EVs-treated cells. (D) qRT-PCR detection of Lin28b expression in the SW480 and SW620 cells transfected with OE-Lin28b. ∗p < 0.05 versus OE-NC-transfected cells. (E) Western blot assay of the protein expression of Lin28b in the SW480 and SW620 cells transfected with OE-Lin28b. ∗p < 0.05 versus OE-NC-transfected cells. (F) qRT-PCR detection of the expression of NRP-1 in the SW480 and SW620 cells transfected with OE-NRP-1. ∗p < 0.05 versus OE-NC-transfected cells. (G) Western blot assay detection of the expression of NRP-1. ∗p < 0.05 versus OE-NC-transfected cells. (H) OE-Lin28b or OE-NRP-1 and OE-SIRT6 were used to treat SW480 and SW620 cells; western blot assay detection of the expression of SIRT6, NRP-1, Lin28b. ∗p < 0.05 versus OE-NC-transfected cells, #p < 0.05 versus OE-SIRT6-transfected cells. (I) ChIP experiment to identify the effect of SIRT6 on the acetylation of Lin28b promoter region. ∗p < 0.05 versus OE-NC-transfected cells. (J) RIP test of the binding of Lin28b to NRP-1. ∗p < 0.05 versus OE-NC-transfected cells. (K) Cell viability detected by CCK-8 assay. ∗p < 0.05 versus OE-NC-treated cells, #p < 0.05 versus OE-SIRT6-treated cells. (L) Cell migration detected by Transwell assay. ∗p < 0.05 versus OE-NC treated cells, #p < 0.05 versus OE-SIRT6 treated cells. (M) Cell invasion detected by Transwell assay. In (K)–(M), SW480 and SW620 cells were transfected with OE-Lin28b or OE-NRP-1 and OE-SIRT6 alone, or co-transfected with OE-SIRT6 and OE-Lin28b, OE-NRP-1, and OE-SIRT6 in combination. ∗p < 0.05 versus OE-NC-transfected cells, #p < 0.05 versus OE-SIRT6-transfected cells. The data were measurement data, expressed as mean ± SD. The unpaired t test was used for analysis between the two groups, one-way analysis of variance was used for comparison among multiple groups, and two-way analysis of variance was used for comparison between different groups at different time points.

To verify whether SIRT6 affects the occurrence and transfer of CRC by affecting the Lin28b/NRP-1 axis, we overexpressed SIRT6 in SW480 and SW620 cells and measured the SIRT6, Lin28b, and NRP-1 protein expression by western blot assay. Results showed that overexpression of SIRT6 inhibited Lin28b and NRP-1 expression (Figure 5A). SW480 and SW620 cells were co-cultured with NCM460-EVs and HCT116-EVs, respectively. Results from this treatment showed that miR-25, Lin28b, and NRP-1 were upregulated by co-culture with HCT116-EVs relative to NCM460-EVs, whereas SIRT6 was downregulated (Figures 5B and 5C). Hence, we concluded that miR-25 transferred by EVs could upregulate Lin28b and NRP-1 expression by inhibiting SIRT6.

Next, Lin28b and NRP-1 overexpression plasmids (OE-Lin28b and OE-NRP-1) were constructed to further investigate their roles in CRC. qRT-PCR and western blot assay results showed that OE-Lin28b and OE-NRP-1 could increase Lin28b and NRP-1 expression in SW480 and SW620 cells (Figures 5D–5G). SW480 and SW620 cells were transfected with OE-Lin28b or OE-NRP-1 and OE-SIRT6 alone or co-transfected with OE-SIRT6 and OE-Lin28b, OE-NRP-1, and OE-SIRT6 in combination. As shown by western blot assay (Figure 5H), upregulation of SIRT6 suppressed Lin28b and NRP-1 expression, while upregulation of Lin28b increased NRP-1 expression. Restoration of Lin28b rescued the NRP-1 expression inhibited by OE-SIRT6, and the NRP-1 expression was increased by OE-NRP-1 in the presence of OE-SIRT6. A chromatin immunoprecipitation (ChIP) assay was performed to verify the deacetylation effect of SIRT6 on the Lin28b promoter (Figure 5I). This experiment showed that overexpression of SIRT6 in two different CRC cell lines significantly reduced deacetylation of H3K56Ac and H3K9Ac in the Lin28b promoter region. Thus, acetylation of the Lin28b promoter region by SIRT6 was achieved at H3K56Ac and H3K9Ac sites. The RNA immunoprecipitation (RIP) experiment verified the binding of Lin28b to NRP-1 (Figure 5J). As depicted in Figures 5K–5N, OE-SIRT6 inhibited growth and migration and invasive potentials of SW480 and SW620 cells, while OE-Lin28b and OE-NRP-1 promoted those potentials. Restoration of Lin28b and NRP-1 reversed the suppressive effects of SIRT6 on the growth and migration and invasive potentials. Taken together, SIRT6 could inhibit CRC cell viability and migration by inhibiting Lin28b and NRP-1.

### miR-25 from CRC cell-derived EVs inhibits SIRT6 to promote the tumor metastasis *in vivo*

**Figure 6:**
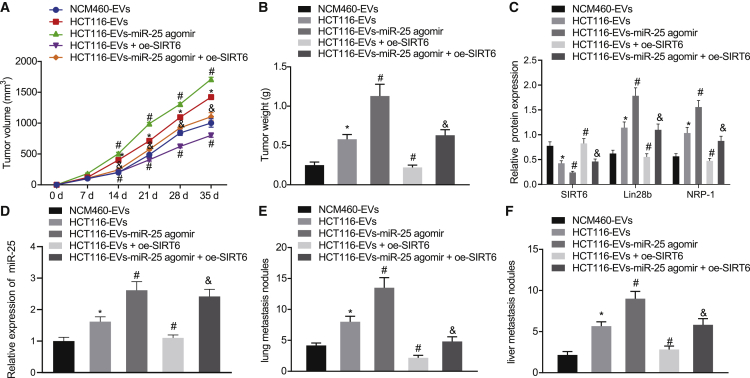
miR-25 in CRC cell-derived EVs promotes the tumorigenic and metastatic potentials of CRC cells *in vivo* through inhibiting SIRT6 SW620 cells were infected with lentivirus expressing OE-SIRT6 and treated with HCT116-EVs/HCT116-EVs-miR-25 agomir, while non-infected SW620 cells were treated with NCM460-EVs/HCT116-EVs/HCT116-EVs-miR-25 agomir. The SW620 cell suspension was subcutaneously injected into nude mice. (A) Tumor growth curves showing tumor volume. (B) Weight of the tumors formed in nude mice. (C) SIRT6, Lin28b, and NRP-1 expression in tumor tissues measured by western blot assay. (D) qRT-PCR detection of the expression of miR-25 in the tumor tissues. (E) H&E staining of the mouse lung tissues to observe the metastasis. (F) H&E staining of the mouse liver tissues to observe the metastasis. ∗p < 0.05 versus NCM460-EVs group, #p < 0.05 versus HCT116-EVs group, &p < 0.05 versus HCT116-EVs-miR-25 agomir group. The data were measurement data, expressed as mean ± SD. The data among multiple groups were analyzed by one-way analysis of variance, and comparison of tumor volume at different time points was performed using repeated-measures analysis of variance; n = 6.

To further substantiate the role of EVs-containing miR-25 in tumor growth and metastasis *in vivo*, SW620 cells were infected with lentivirus expressing OE-SIRT6 and treated with HCT116-EVs/HCT116-EVs-miR-25 agomir, while non-infected SW620 cells were treated with NCM460-EVs/HCT116-EVs/HCT116-EVs-miR-25 agomir. The SW620 cell suspension was subcutaneously injected into nude mice, and the resultant tumor volume was measured every 7 days to calculate the growth curve. After 5 weeks, the mice were euthanized, and the tumor was removed for analysis. As shown in Figures 6A and 6B, compared with NCM460-EVs, treatment with HCT116-EVs increased tumor growth rate and weight, whereas HCT116-EVs-miR-25 agomir treatment had relatively large effects in elevating tumor growth rate and weight. However, OE-SIRT6 infection partially inhibited tumor growth rate and reduced tumor weight, which were enhanced by HCT116-EVs and HCT116-EVs-miR-25 agomir.

The expression of miR-25, SIRT6, Lin28b, and NRP-1 in tumor tissues was measured by western blot assay and qRT-PCR (Figures 6C and 6D). Compared with NCM460-EVs treatment, HCT116-EVs increased the expression of miR-25, Lin28b, and NRP-1 and inhibited the expression of SIRT6. Treatment with HCT116-EVs-miR-25 agomir also increased the expression of miR-25, Lin28b, and NRP-1 and suppressed the expression of SIRT6 relative to HCT116-EVs. However, the changes in the aforementioned factors caused by treatment with HCT116-EVs or HCT116-EVs-miR-25 agomir were partially reversed by restoration of SIRT6. The hematoxylin and eosin (H&E) staining results (Figures 6E and 6F) revealed that, compared with NCM460-EVs, HCT116-EVs promoted lung and liver metastasis. HCT116-EVs-miR-25 agomir led to an enhanced promotion of lung and liver metastasis compared to the HCT116-EVs treatment. However, the promotive effects of HCT116-EVs or HCT116-EVs-miR-25 agomir on lung and liver metastasis were partially counteracted by restoration of SIRT6. Therefore, EV-shuttled miR-25 from CRC cells could promote tumorigenesis and metastasis of CRC through downregulation of SIRT6.

## Discussion

CRC is a heterogeneous disease that is one of the major causes of cancer-related mortality worldwide,^21^ with tumor metastasis being the leading cause of mortality of CRC patients,^22^ which calls for new interventions to discourage metastasis. In this study, we investigated the molecular mechanism of EVs derived from CRC cells to affect CRC growth and metastasis. Results provided evidence that miR-25 in EVs derived from CRC cells could expedite CRC development and metastasis through targeting histone deacetylase SIRT6 expression and activating the Lin28b/NRP-1 axis.

Our study revealed that the EVs secreted from CRC cells promoted the viability, migration, and invasion of CRC cells. CRC cell-derived EV-enriched proteins serve as markers of metastatic cancer and are involved in cancer progression.^23^ EVs can promote the progression of CRC through transferring mutant β-catenin to recipient cells.^24^ EVs can also carry cargos consisting of oncoproteins, oncopeptides, and miRNAs from donor to recipient cells to make changes in the tumor microenvironment.^25^ For example, colon cancer cell-derived EVs contained abundant miR-92a-3p, which plays pro-metastatic functions in colon cancer.^26^ In addition, we found that miR-25 was upregulated in CRC tissues and cell lines and that its high expression was associated with metastatic CRC. Similar to our present findings, others report that miRNAs originating from tumor cells may work as non-invasive biomarkers for detecting CRC.^27^ miR-25, which belongs to the oncogenic miR-106b-25 cluster, was found to be upregulated in CRC stromal tissues as compared to normal stroma.^9^ Highly expressed miR-25 is present in CRC and is closely related to the progression of tumor.^9^ Furthermore, miR-25 has been proposed to be a tumor promoter in gastrointestinal cancers, such as gastric cancer,^28^, ^29^, ^30^ and was documented to be enriched in the EVs secreted from CRC cells and to be transferred between cancer cells. Interestingly, cancer-derived exosomes (a major type of EVs) transfer miR-25 to drive tumor-induced pre-metastatic niche formation in CRC.^10^ Hence, we speculated that CRC cell-derived EVs might transfer a cargo of miR-25 and consequently accelerate CRC progression *in vitro* and tumor metastasis *in vivo*, which was confirmed by our findings in a co-culture system.

Another important finding of this study was that miR-25 could target SIRT6 and downregulate SIRT6 expression. SIRT6 overexpression could impede the growth, migration, and invasiveness of CRC cells. Consistent with our findings, SIRT6 functioned as an anti-oncogene in CRC through inhibiting cancer stem cell proliferation.^31^ Also, SIRT6 retarded the malignant progression of colon cancer via mediating PTEN/AKT signaling,^12^ and SIRT6 has been reported to be a tumor-suppressive gene in other types of human cancer. For example, SIRT6 inhibits the JAK2/STAT3 pathway to suppress the growth of gastric cancer.^32^ SIRT6 acts as a tumor suppressor in glioma through inhibiting the expression of the RNA-binding protein PCBP2.^33^ By conducting rescue experiments, we found that miR-25 delivered by EVs could inhibit the expression of SIRT6 and enhance the malignant phenotypes and tumorigenicity of CRC cells. As mentioned above, SIRT6 functions as an anti-oncogene by mediating different signaling factors. The present research further investigated the downstream mechanism associated with the anti-proliferative and anti-migratory functions of miR-25-targeted inhibition of SIRT6.

The present findings also suggested that SIRT6 could downregulate NRP-1 by inhibiting Lin28b, thereby impeding the proliferative, migratory, and invasive abilities of CRC cells. Moreover, overexpression of SIRT6 in EVs derived from HCT116 cells suppressed lung and liver metastases. In line with our findings, the inactivation of SIRT6 could promote the progression of pancreatic ductal adenocarcinoma and metastasis through increasing Lin28b.^16^ The promotive effects of Lin28b on gastric cancer stemness can be diminished by deleting NRP-1.^18^ Furthermore, overexpressed Lin28b can promote colon cancerogenesis by activating B cell lymphoma 2.^34^ NRP-1 is highly expressed in a subset of high-grade precursor lesions and in gastrointestinal adenocarcinomas.^35^ Moreover, NRP-1 can promote the migration and survival of colon cancer cells in response to vascular endothelial growth factor (VEGF) binding.^36^

**Figure 7:**
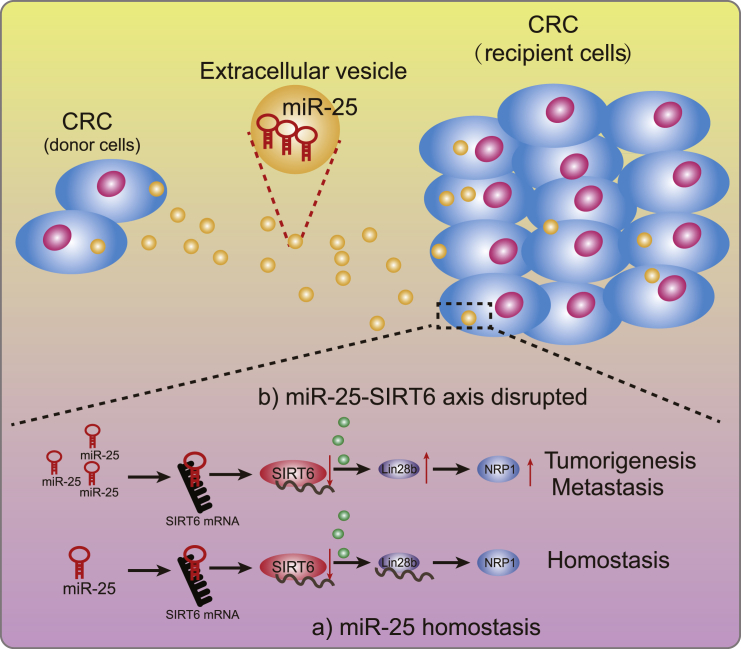
The mechanism graph of the regulatory network and function of miR-25 shuttled by CRC cell-derived EVs in CRC In CRC cell-derived EVs, miR-25 targets and inhibits the expression of SIRT6, while SIRT6 inhibits the expression of Lin28b/NRP-1, leading to suppressed viability, migration, and invasion of CRC cells. Thus, CRC cell-derived EVs loaded with high miR-25 expression promote the tumorigenesis and metastasis of CRC cells.

The above findings demonstrated that EVs derived from CRC transferred miR-25 to tumor cells to downregulate Lin28b and NRP-1 by targeting SIRT6, thereby promoting the metastasis of CRC (Figure 7). Although the clinical utility of these observations remains to be established, the present experimental results lay a theoretical foundation for in-depth understanding of CRC mechanisms and for developing new treatment methods. However, more experiments with the use of new technology should be performed to validate the functional delivery of miR-25 via EVs but not other components in EVs. Also, the mechanism of miR-25 entering the EVs and recipient cells remains to be established, and the release and internalization of EVs need to be investigated in the future.

## Materials and methods

### Ethics statement

The study was approved by the Ethics Committee of Linyi People’s Hospital (Linyi, Shandong, P.R. China), with informed consent signed by each participant. This experimental procedure and animal use protocol had been approved by the Animal Ethics Committee of Linyi People’s Hospital (Linyi, Shandong, P.R. China).

### Patient enrollment

Biopsy cancerous tissues and non-cancerous tissues adjacent to cancerous tissues were collected from 50 patients with CRC who underwent resection in Linyi People’s Hospital (Linyi, Shandong, P.R. China) from 2017 to 2018. Fresh biopsy tissues were frozen and stored in liquid nitrogen. None of the enrolled participants had received radiotherapy or chemotherapy prior to surgery. Based on the patient’s medical history, TNM staging information was recorded (T, primary tumor; N, regional lymph node; M, distant metastasis).

### Cell culture

Human colon cell line NCM460, and human CRC cell lines SW480, SW620, LOVO, and HCT116 were purchased from the American Type Culture Collection (Manassas, VA, USA). All these cell lines were cultured in Dulbecco’s modified Eagle’s medium (DMEM) (GIBCO, Gaithersburg, MD, USA) with 10% fetal bovine serum (FBS) (HyClone, Logan, UT, USA) and 1% penicillin/streptomycin (Invitrogen, Carlsbad, CA, USA) at 37°C and in 5% CO_2_.

### Extraction and identification of EVs

Equal numbers of NCM460 and HCT116 cells were placed in a 10 cm Petri dish and cultured in DMEM with EV-depleted serum. After 48 h, conditioned medium (CM) was collected and filtered through a 0.22 μm filter (Merck Millipore, Billerica, MA, USA). EVs in CM were separated by ultracentrifugation using the Optima Max-XP instrument (Beckman Coulter, CA, USA). The isolated EVs were observed under a Philips CM120 BioTwin TEM (FEI, Hillsboro, OR, USA). A Nanosizer instrument (Malvern Instruments, Malvern, UK) Dynamic light scattering (DLS) was utilized to measure the size and distribution of EVs. Western blot assay was performed to identify the characteristics of EVs by detecting EV-specific surface markers CD63 (1:2,000, Abcam, UK, ab216130, rabbit antibody), CD9 (1:1,000, Abcam, UK, ab236630, rabbit antibody), CD81 (1:10,000, Abcam, UK, ab109201, rabbit antibody), and endoplasmic reticulum marker Calnexin (1:100,000, Abcam, UK, ab92573, rabbit antibody).

### Internalization of EVs

Purified EVs were labeled with the green fluorescent dye PKH67 (Sigma-Aldrich Chemical Company, St. Louis, MO, USA) according to the manufacturer’s instructions. In detail, cells were seeded in 8-well chamber slides (Thermo Fisher Scientific, Waltham, MA, USA) (8,000 cells/well), and then 5 μL of PKH67 was added for a 24-h incubation. Finally, the cells were washed twice with phosphate-buffered saline (PBS), and the cells were fixed in 4% paraformaldehyde (Leagene, Beijing, P.R. China) for 15 min. Nuclei were stained with 4′,6-diamidino-2-phenylindole (0.5 mg/mL, Invitrogen, Carlsbad, CA, USA), and images were taken using a Zeiss LSM 780 confocal microscope (Zeiss, Jena, Germany).

### Cell transfection

NCM460, HCT116, SW480, and SW620 cells in logarithmic phase were collected and seeded in 6-well plates at 1 × 10^5^ cells per well and were routinely cultured for 24 h. After the cell confluence reached about 75%, transfection was performed according to instructions in the Lipofectamine 2000 kit (Invitrogen, Carlsbad, CA, USA). miR-25 mimic, miR-25 inhibitor, mimic-NC, inhibitor-NC, miR-25 agomir, and agomir-NC were all purchased from RiboBio (Guangzhou, P.R. China), referring to the plasmid concentrations indicated by the manufacturer. Overexpression plasmids (OE-SIRT6, OE-Lin28b, and OE-NRP-1), with pGL3-empty vector as backbone, control plasmids, and lentiviral vectors for packaging OE-SIRT6 were purchased from GeneCopoeia (Rockville, MD, USA). NCM460 and HCT116 cells were transfected with miR-25 mimic, mimic-NC, miR-25 inhibitor, and inhibitor-NC, respectively. SW480 and SW620 cells were transfected with miR-25 mimic, mimic-NC, miR-25 inhibitor, inhibitor-NC, OE-NC, OE-SIRT6, OE-Lin28b, OE-NRP-1, OE-SIRT6 + OE-Lin28b, and OE-SIRT6 + NRP-1 in combination, respectively. EVs were extracted from the HCT116 cells transfected with miR-25 mimic and mimic-NC and were designated as EVs-miR-25 mimic and EVs-mimic-NC, respectively. The SW480 and SW620 cells transfected with OE-NC or OE-SIRT6 were co-cultured with the EVs-miR-25 mimic and EVs-mimic-NC. For experiments *in vivo*, EVs were extracted from the NCM460 cells, HCT116 cells, and HCT116 cells treated with miR-25 agomir and were designated as NCM460-EVs, HCT116-EVs, and HCT116-EVs-miR-25 agomir, respectively. Next, SW480 cells were co-cultured with the NCM460-EVs, HCT116-EVs, and HCT116-EVs-miR-25 agomir alone or co-treated with HCT116-EVs + OE-SIRT6 and HCT116-EVs-miR-25 agomir + OE-SIRT6 together.

### CCK-8 assay

CCK-8 (Dojindo, Kyushu Island, Japan) was utilized for detection of cell viability. The cells were seeded in 96-well plates (5 × 10^3^ cells/well). On days 1, 2, 3, 4, and 5, 10 μL of CCK-8 solution and 100 μL of fresh medium were loaded to each well for incubation at 37°C for 1 h. Absorbance was detected at 450 nm using a Bio-Rad 680 microplate reader (Bio-Rad, Hercules, CA, USA).

### Transwell assay

The apical chamber surface of the bottom membrane of Transwell chamber (8 mm pore size; Corning Star, Cambridge, MA, USA) was coated with Matrigel from BD Biosciences (San Jose, CA, USA) for Transwell invasion assays, and the Transwell chamber without Matrigel was employed for Transwell migration assays. The cells were cultured in serum-free medium for 12 h and then harvested and resuspended in serum-free medium (1 × 10^5^ cells/mL). 10% FBS was added to the basolateral chamber, and 100 μL of the cell suspension was added to the apical Transwell chamber and incubated together at 37°C. After 24 h, the cells were fixed with 100% methanol and stained with 1% toluidine blue (Sigma-Aldrich Chemical, St. Louis, MO, USA). The stained invading cells were counted using an inverted light microscope (Carl Zeiss, Jena, Germany).

### qRT-PCR

Total RNA was extracted from cells using TRIzol Reagent (Gibco, Carlsbad, CA, USA), and 1 μg total RNA (mRNA) was reverse-transcribed into cDNA using Revert Aid first-strand cDNA synthesis kit (Fermentas, Thermo Fisher Scientific). The real-time qPCR was performed with SYBR Premix ExTaqTM II in an ABI PRISM 7900HT System (ABI, Thermo Fisher Scientific), with three replicates set for each well. The mRNA expression relative to glyceraldehyde-3-phosphate dehydrogenase (GAPDH) was determined using the 2^−ΔΔCT^ method. The PCR primers are shown in Table 1. The SeraMir Exosome RNA Purification Kit (System Biosciences, Mountain View, CA, USA) was utilized to isolate EVs-miRNA with cel-miR-39-3p as the reference. The PureLink miRNA Isolation Kit (Invitrogen, Carlsbad, CA, USA) was employed to extract cellular miRNA, with U6 as reference. The RNA (miRNA) was reversed to cDNA using TaqMan microRNA assay kit (Applied Biosystems, Foster City, CA, USA). The universal reverse primers, provided by FastStart Universal SYBR Green Master Mix (Roche, Indianapolis, IN, USA) and TaqMan microRNA assay kit, were used in qPCR.

**Table 1:** Primer sequences for qRT-PCR

Genes	Primer sequences
miR-25-3p	F: 5′-TTGCACTTGTCTCGTCTGA-3′
	R: 5′-GTGCAGGGTCCGAGGT-3′
Cel-miR-39-3p	F: 5′-UCACCGGGUGUAAUCAGCUUG-3′
	R: 5′-AACGCTTCACGA ATTTGCGT-3′
SIRT6	F: 5′-AAATAACTAAAGCCCGCCTC-3′
	R: 5′-TCCTGAGATGATGACTATGTG-3′
Lin28b	F: 5′-GCCCCTTGGATATTCCAGTC-3′
	R: 5′-TGACTCAAGGCCTTTGGAAG-3′
NRP-1	F: 5′-CATTGCTCGTTCCCCTCCTT-3′
	R: 5′-TGTTTCTGGACCCGTTGGAG-3′
GAPDH	F: 5′-GGAGCGAGATCCCTCCAAAAT-3′
	R: 5′-GGCTGTTGTCATACTTCTCATGG-3′
U6	F: 5′-CTCGCTTCGGCAGCACA-3′
	R: 5′-AACGCTTCACGAATTTGCGT-3′

qRT-PCR, quantitative reverse-transcriptase polymerase chain reaction; miR-25-3p, microRNA-25-3p; Cel-miR-39-3p, *Caenorhabditis elegans* microRNA-39-3p; SIRT6, sirtuin 6; Lin28b, lin-28 homolog B; NRP-1, neuropilin-1; GAPDH, glyceraldehyde-3-phosphate dehydrogenase; F, forward; R, reverse.

### Western blot assay

The cultured cells were collected and lysed with enhanced radioimmunoprecipitation assay lysis buffer (Boster Biological Technology, Wuhan, P.R. China) containing protease inhibitors. The bicinchoninic acid kit (Boster Biological Technology) was utilized to determine the protein concentration. Proteins were separated with 10% sodium dodecyl sulfate-polyacrylamide gel electrophoresis, the separated proteins were electro-transferred to polyvinylidene fluoride membrane (Immobilon P, Millipore, Billerica, MA, USA), and the membrane was treated with 5% skimmed milk at ambient temperature for 2 h to block non-specific binding. The membrane was incubated with primary antibody at 4°C overnight and then with horseradish peroxidase conjugated secondary antibody at 37°C for 1 h. Enhanced chemiluminescence reagent (Thermo Fisher Scientific) was utilized to visualize the immunoreactive bands, followed by imaging using ChemiDoc XRS Plus luminescent image analyzer (Bio-Rad, Hercules, CA, USA). ImageJ analysis software was utilized to quantify the gray value of protein bands. Antibodies included SIRT6 (A7416, 1:2,000, ABclonal, Woburn, MA, USA), NRP-1 (A19087, 1:2,000, ABclonal), GAPDH (AC033, 1:50,000, ABclonal), Lin28B (ab191881, 1:2,000, abcam), rabbit secondary antibody (AS014, 1:10,000, ABclonal), and murine secondary antibody (AS003, 1:10,000, ABclonal).

### Dual-luciferase reporter gene assay

The synthetic SIRT6 3′UTR gene fragment was introduced into pGL3-control vector (Promega, Madison, WI, USA) using the endonuclease site, and the complementary sequence mutation site of the seed sequence was introduced on the SIRT6 WT through restriction enzyme treatment. After digestion, T4 DNA ligase was used to insert the target fragment into the pGL3-control vector. The MUT SIRT6 plasmid was generated with the binding sites mutated and was inserted into the pGL3-control vector using T4 DNA ligase. The WT and MUT luciferase reporter plasmids that showed the correct sequences were co-transfected with miR-25 mimic into HEK293T cells (Cell Resource Center, Shanghai Institute of Life Sciences, Chinese Academy of Sciences, Shanghai, P.R. China). After 48 h of transfection, cells were collected and lysed, and the luciferase activity was measured using the Dual-Luciferase Reporter Assay System kit (Promega, Madison, WI, USA) and TD-20/20 luminometer (E5311, Promega, Madison, WI, USA).

### ChIP

After the cell confluence reached about 70%–80%, the cells were fixed with 1% formaldehyde at ambient temperature for 10 min. After cross-linking, they were randomly broken by ultrasonication, with 15 cycles of 10 s of ultrasound at 10 s intervals. The cells were centrifuged at 13,000 rpm at 4°C. The supernatant was incubated with antibodies: rabbit anti-immunoglobulin G (IgG) (ab109489, 1:100, Abcam, Cambridge, UK), anti-H3K9Ac (Millipore 07-352, Billerica, MA, USA), and anti-H3K56Ac (ab76307, Abcam, Cambridge, UK) overnight at 4°C. The endogenous DNA-protein complex was precipitated with protein agarose/Sepharose, the supernatant was discarded after a short centrifugation, and the non-specific complex was washed. After de-crosslinking at 65°C overnight, DNA fragments were harvested by phenol/chloroform extraction for measuring enrichment of H3K9Ac and H3K56Ac in Lin28b promoter fragment-specific primers.

### RIP assay

The cells were lysed by 100 U/mL ribonuclease inhibitor (R0102-10kU, Beyotime Institute of Biotechnology, Shanghai, P.R. China) and 25 mM Tris-HCl buffer (pH 7.5). The protein-A/G agarose beads (Piells, Thermo Fisher Scientific) were pre-coated with 3 μg anti-Lin28b antibody (AB191881, Abcam, Cambridge, UK) or rabbit IgG (A7016, Beyotime, Shanghai, P.R. China). After that, the cells were incubated with the beads at 4°C for 1.5 h.

### Tumor formation and metastasis in murine model

The 4- to 6-week-old healthy BALB/c-nu/nu nude mice were purchased from the Department of Pharmacology, Institute of Materia Medica, Chinese Academy of Medical Sciences (Beijing, P.R. China). The nude mice were caged in specific-pathogen-free animal laboratory with room humidity of 60%–65%, temperature of 22°C–25°C, and free food and water under a 12 h light and dark cycle. Mouse experiments started after 1 week of adaptive feeding, with monitoring of their health status before the experiment. Each mouse was subcutaneously injected with 0.2 mL SW620 cell suspension (1 × 10^7^ cells/mL). The inoculation site was observed periodically, and the tumor volume was calculated from the long (A) and short (B) diameters measured using a Vernier caliper. The tumor volume was calculated as V = AB^2^/2. After 5 weeks, the mice were euthanized and the tumors were isolated for recording the tumor size and weight and for detection of the expression of miR-25, SIRT6, Lin28b, and NRP-1 in the tumor tissues.

Tail vein and intrasplenic injection of tumor cells was performed to construct mouse models of lung metastasis and liver metastasis, respectively. For the lung metastasis model, 2 × 10^6^ treated SW620 cells were injected into the tail vein of nude mice. After 30 d, the mice were euthanized by an overdose of pentobarbital sodium (35–40 mg/kg), and the lungs were isolated for examination. For establishing the liver metastasis model, the nude mice were anesthetized with pentobarbital sodium (35–40 mg/kg), and the spleen was excised by laparotomy. Then, a total of 2 × 10^6^ treated SW620 cells were injected into the spleen sac. After 30 d, the mice were euthanized, and the liver was collected for examination.

Mouse tumor tissues and lung and liver tissues were stained with H&E staining kit (Beyotime). A Leica microscope (Wetzlar, Germany) was utilized to observe tumor tissue morphology and lung and liver metastasis.

### Statistical analysis

The statistical analysis of data was performed using SPSS 21.0 (IBM, Armonk, NY, USA) statistical software. The measurement data were expressed in the form of mean ± SD. The comparison between cancer tissues and non-cancerous tissues was performed by paired t test. The comparison between other two groups of data was analyzed by unpaired t test. The comparison among multiple groups was carried out by one-way analysis of variance. Two-way analysis of variance was utilized to compare cell viability at different time points, and tumor volume at different time points was compared using repeated-measures analysis of variance. Pearson’s correlation analysis was performed to analyze the relationship between SIRT6 and miR-25. p <0.05 indicated that the difference was statistically significant.

### Availability of data and materials

The datasets generated and/or analyzed during the current study are available from the corresponding author on reasonable request.

## Supplemental information

Document S1. Figure S1

Document S2. Article plus Supplemental information
