# Supplementary material for: Transfer of microRNA-25 by colorectal cancer cell-derived extracellular vesicles facilitates colorectal cancer development and metastasis
Source: Mol Ther Nucleic Acids. 2020 Nov 26;23:552–64. doi: 10.1016/j.omtn.2020.11.018 (PMC7810909; doi:10.1016/j.omtn.2020.11.018)
Supplement: Document S1. Figure S1 [file mmc1.pdf]

OMTN, Volume *23*

Supplemental Information

Transfer of microRNA-25 by colorectal cancer cell-derived extracellular vesicles facilitates colorectal cancer development and metastasis

Shanchao Wang, Zeyan Zhang, and Qianfu Gao

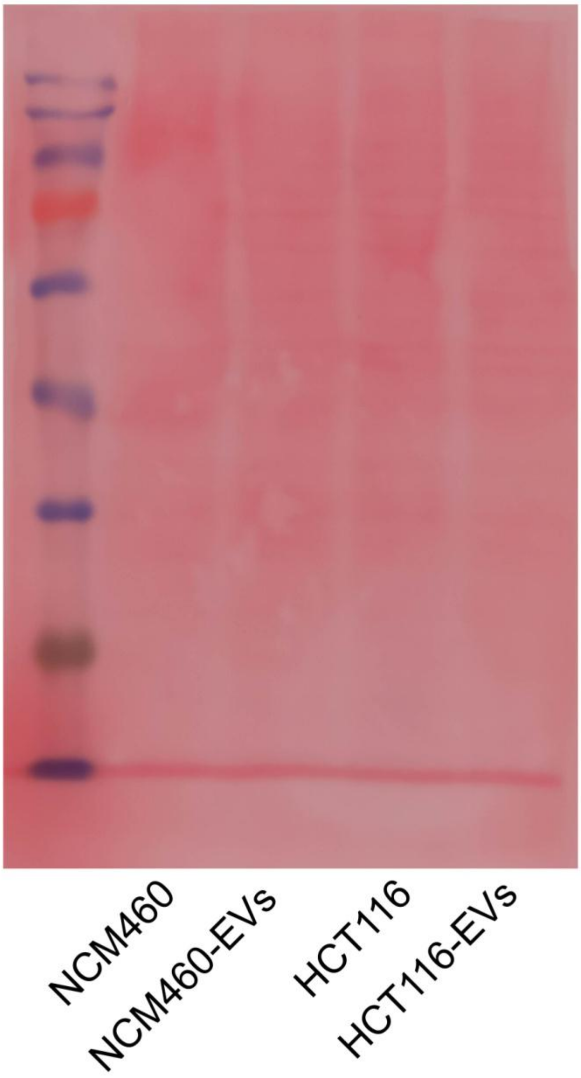

Supplementary Figure 1 The polyvinylidene fluoride membrane was stained with Ponceau red dye to ensure consistent sample size before the measurement of EV markers in Figure 1E.
